# Highly Stable, Bending-Tolerant, and Sustainable Flexible Heater through a Scalable Papermaking Procedure

**DOI:** 10.3390/ma17143507

**Published:** 2024-07-15

**Authors:** Jiajie Liu, Huacui Xiang, Wei Wang, Xiujuan Tao, Zhou Bai, Zhijian Li, Haiwei Wu, Suochao Yuan, Hongwei Zhou, Hanbin Liu

**Affiliations:** 1 College of Chemistry and Chemical Engineering, Shaanxi University of Science & Technology, Xi’an 710021, China; 2 Shaanxi Provincial Key Laboratory of Papermaking Technology and Specialty Paper Development, College of Bioresource Chemical and Materials Engineering, Shaanxi University of Science & Technology, Xi’an 710021, China; 3 School of Materials and Chemical Engineering, Xi’an Technological University, Xi’an 710021, China

**Keywords:** flexible heater, graphite nanoplates, cellulose fiber, papermaking, scalable

## Abstract

Flexible electrothermal heaters have attracted abundant attention in recent years due to their wide applications, but their preparation with high efficiency remains a challenge. Here in this work, a highly stable and bending-tolerant flexible heater was fabricated with graphite nanosheets and cellulose fibers through a scalable papermaking procedure. Its electrothermal property can be enhanced by a hot-pressing treatment and introduction of cationic polyacrylamide (CPAM) during the papermaking protocol. The flexible heater may quickly reach its maximum temperature of 239.8 °C in around 1 min at a voltage of 9 V. The power density was up to 375.3 °C cm^2^ w^−1^. It appeared to have a high tolerance for bending deformation with various curvatures, and the temperature remained stable even under 100 bending with frequency of around 0.17 Hz. Over 100 alternatively heating and cooling cycles, it worked stably as well. It was proved to be used as wearable heating equipment, soft heaters, and aircraft deicing devices, suggesting its great prospect in the field of heat management.

## 1. Introduction

Flexible electrothermal heaters transfer electrical energy into thermal energy depending on the mechanism of Joule heating [1]. They have attracted more and more attention in recent years due to their great prospects in versatile applications including touch pads [2], display devices [3], sensors [4,5], electric vehicles [6,7], wearable equipment [1,8,9], deicing for aircrafts [10,11,12], and so on. The industrial applications may be hindered by the scalability of the production of the heating materials. In addition, the working stability under deformation may be another important issue for flexible heating devices. However, the scalable preparation of the electrothermal materials and how to keep the working stability of the heaters under deformation remains a challenge [6,13,14,15].

Most reported strategies to target the scalable fabrication of flexible heaters include composition with polymers [8,13,16,17,18,19] and printing or coating of nanomaterials [3,5,18,19,20,21,22,23,24,25,26,27,28,29] onto the soft substrates. Good electrothermal performance might be achieved, but the applications of these devices may be restricted by the high price of the nanowires or the poor flexibility of the substrates. The papermaking technique invented one thousand years ago has been developed into a modern technology with super high efficiency [30,31,32]. The paper-based flexible heaters prepared through the papermaking technology may achieve a self-supported electrothermal device with low cost and high possibility for large-scale production. In addition, the raw materials for papermaking are cellulose fibers that are from the renewable source of wood or grasses, and the devices may be degraded in the natural environment being free of pollution [16]. Therefore, this kind of flexible heater might be sustainable and perfectly matches the requirements of the green development of the present world [28,33].

**Figure 1:**
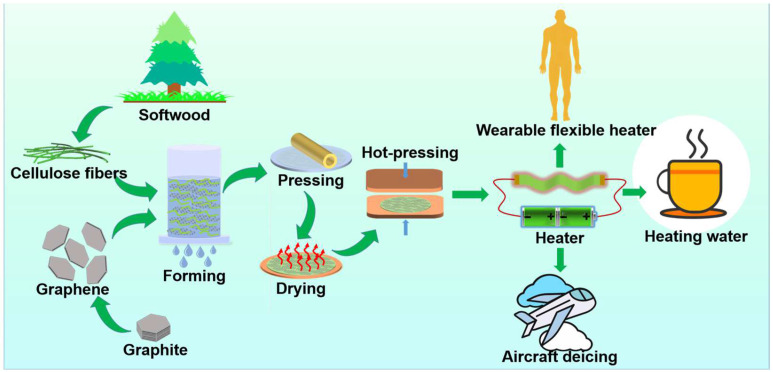
Schematic diagram of preparation and application of the flexible heater.

In this work, a highly stable, bending-tolerant, and sustainable flexible heater was prepared through the papermaking procedure using cellulose fibers and graphite nanoplates (Figure 1). The electrothermal property was improved by the followed hot-pressing treatment and adding cationic polyacrylamide (CPAM) during the papermaking procedure. The electrothermal heating performance was investigated under various voltages, and its work stability was also valuated. It was found to be used in wearable heaters, water heating, and deicing as well, suggesting its versatile applications in wearable equipment and protection of aircraft and smart devices that requires thermal management.

## 2. Experimental Part

### 2.1. Materials

A softwood pulp sample was supplied by Fujian Qingshan Paper Industry Co., Ltd. (Fujian, China). The graphite nanoplates slurry (5 wt% in water) was purchased from XFNANO Materials Tech Co., Ltd. (Nanjing, China), which was mechanically exfoliated from natural graphite in flakes. The CPAM (${\bar{M}}_{n}$ ≈ 1.2 × 10^7^ g mol^−1^) was purchased from Guangya Disinfection Products Co., Ltd. (Henan, China). Deionized (DI) water was treated and collected with the Milli-Q Integral Water Purification System (15 MΩ·cm).

### 2.2. Preparation of the Composite Paper through a Papermaking Procedure

The composite paper with cellulose fibers and graphite nanoplates in this work was prepared using a typical lab-scale papermaking protocol [30]. At the beginning, 30 g of softwood pulp board was soaked in water for 4 h then defibered in water with a disperser for 4000 turns, yielding cellulose fibers. The fibers were then compressed to remove excess water to achieve a water content of 89 wt%. Secondly, the papermaking technique in the lab was performed, setting a basis weight of 80 g m^−2^ with various contents of graphite nanoplate. Taking the paper with 22 wt% graphite nanoplates as an example, a typical protocol was carried out as follows. A total of 11.2 g of graphite nanoplate slurry (5 wt%, equal to 0.56 g of graphite nanoplates) was added into 200 mL of DI water with ultrasonic treatment for 5 min. Then, 18.17 g of wet cellulose fibers (equivalent to 1.98 g of pure fibers) and an additional 2000 mL of water were mixed into the above graphite nanoplate slurry to yield a paper pulp. Different amounts of CPAM (0, 0.2 wt%, 0.4 wt%, 0.6 wt%, 0.8 wt%, relative to absolute dry fibers) were added. A piece of the composite paper with a diameter of 20 cm was then obtained on a lab papermaking machine (Xianyang Tongda Papermaking Equipment Co, Ltd., Xianyang, China) involving three steps, including draining, pressing, and drying, as shown in Figure 1. Thirdly, the yielded paper was hot-pressed for 10 min by a plate vulcanizing machine. The temperature in the hot-pressing treatment was 105 °C, and the pressure was 10 MPa to obtain the composite paper.

### 2.3. Preparation of the Flexible Heater

The composite paper composed of cellulose fibers and graphite nanoplates was cut into strips with a size of 40 × 10 mm and connected with copper wires using silver paste, producing the electrothermal heaters. The characterization and the following performance tests were conducted based on this device unless otherwise stated.

### 2.4. Characterization

The microstructure of the heater was visualized by a scanning electron microscope (SEM, vega-3, TESCAN, Brno, Czech Republic) after spraying gold on the surface. The electrothermal property was tested with a multichannel temperature monitor (DC5508a, Pumei, Hangzhou, China) equipped with direct current (DC) power. The infrared (IR) images were taken with the infrared thermal imaging device (Ti32, Fluke, Everett, WA, USA). For the heating performance under bending, the heaters were fixed on a vernier caliper to control their curvature, and the IR images were taken by the Ti32 device. For the performance under twisting, one end of the heater was fixed and the other was twisted. The thermogravimetric analysis (TG) was conducted with a simultaneous thermal analyzer (STA449F3-1053-M, NETZSCH, Bayern, Germany) with a heat rate of 10 °C min^−1^. An electronic tensile testing machine (AI-7000-NGD, Goodtechwil, Qingdao, China) was used to test the mechanical property of the sensor.

## 3. Results and Discussion

The preparation protocol of the flexible heater is illustrated in Figure 1. The cellulose fibers were derived from softwood and the graphite nanoplates came from the graphite. Then, the heater was fabricated through a papermaking procedure using a lab-scale papermaking machine involving steps of draining, pressing, and drying. During the preparation of the cellulose fibers slurry, the CPAM was added to enhance the interaction between the cellulose fibers by hydrogen bonding. After obtaining the paper, a hot-pressing treatment was followed to condense the paper and further strengthen the interactions among cellulose fibers. The addition of CPAM and the hot-pressing treatments were found to improve the electrothermal performance of the flexible heater.

**Figure 2:**
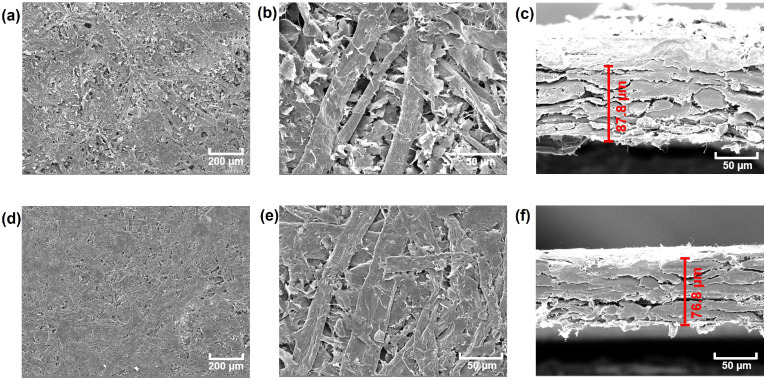
(**a**,**b**) Surface and (**c**) cross-section SEM images of the composite paper with 40% content of graphite before hot pressing. (**d**,**e**) Surface and (**f**) cross-section SEM images of the composite paper with 40% content of graphite after hot-pressing (105 °C, 10 MPa, 10 min).

The obtained composite paper was firstly visualized by SEM. The surface morphology of the composite paper with the content of 40 wt% graphite nanosheets without hot-pressing is shown in Figure 2a, appearing as a smooth and porous plate. In the magnified image of Figure 2b, one may find the cellulose fibers and the lamellar graphite nanosheets. From its cross-section image, it is found that the thickness is around 87.8 μm. After the hot-pressing treatment, the composite paper became much smoother with less pores (Figure 2d,e) compared to the sample before hot-pressing. The stress–strain curve suggested that the tensile strength and elongation at break of the sensor after hot pressing both increased compared to the one without hot pressing (Figure S1). Furthermore, the thickness of the pressed paper decreased to 76.8 μm, suggesting that it was significantly compressed. This confined state of the pressed paper may indicate its good bending tolerance and better conductivity.

**Figure 3:**
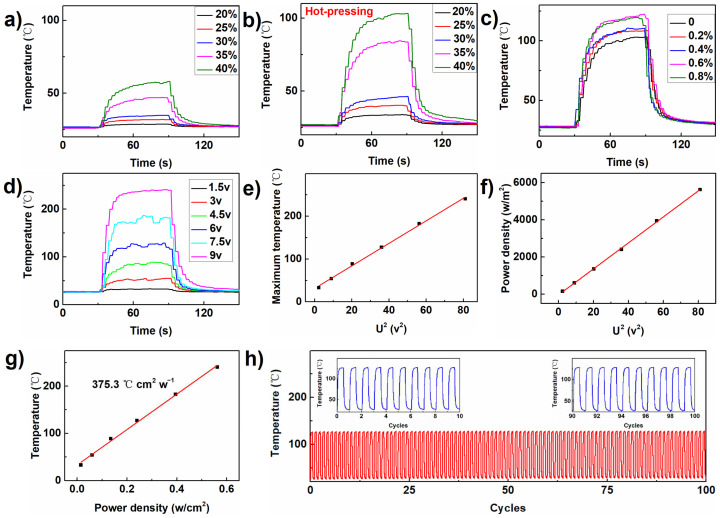
(**a**,**b**) Temperature change curve of the flexible heater with different contents of graphite nanoplates driven by the voltage of 6 V before and after the hot-pressing treatment. (**c**,**d**) Temperature change curve of the flexible heater with different additions of CPAM or under various voltages. The change in (**e**) the maximum temperature and (**f**) power density of the heater with 40 wt% graphite nanoplates as a function of U^2^. The red line in the e-g diagram is a linear fitting curve. (**g**) Changes in the maximum temperature as a function of the power density. (**h**) Temperature of the heater under alternative voltage of 6 V. The red line represents the real-time change of temperature during 100 cycles, and the blue line represents the real-time change of temperature during part of the intercepted time.

The flexible heater was then assembled by connecting copper wires at the two ends of the composite paper, and its electrothermal performance was investigated with the multichannel temperature monitor. The heater was driven with DC power with voltage from 1.5 to 9 V. The temperature change of the flexible heater with different contents of graphite nanoplates driven by the voltage of 6 V was recorded as shown in Figure 3a; it is found that the surface temperature of the heater gradually increases with the extension of time, and it finally targeted a maximum temperature in around 90 s then flowed down when the power was switched off. The temperature jumping process lasted for around 10 s, suggesting a high heating rate. Furthermore, the maximum temperature of the heater was increased from around 28.7 to 58 °C when the content of the graphite nanoplates increased from 20 to 40 wt%. In contrast, the maximum temperature of the heater after the hot-pressing treatment appeared much higher, which shot to around 102.9 °C under 6 V when the content of the graphite nanoplates was 40 wt%, suggesting that the heating property was significantly enhanced through the hot-pressing treatment. The reason may be related to the better connection of the graphite nanoplates in the compressed state, resulting in the increment in the conductive paths in the heater. In the following experiments, the heater with 40 wt% of the graphite nanoplates was used unless otherwise specified.

The impact of the addition of CPAM for the heating performance was then evaluated as shown in Figure 3c. It was found that the maximum temperature at 122.2 °C can be achieved in 90 s when the CPAM addition was 0.6%, which was the highest value with CPAM, and this content was used further in the following studies. The CPAM may generate hydrogen bonding with cellulose fibers, which might be helpful for the condensation of the composite paper and benefit the heating performance. Furthermore, the uniformity and repeatability of such heating performance was investigated. It was found that the heaters using different sites of the composite paper may be heated up to 116 ± 4 °C in around 90 s and the heaters using different batches of the composite paper may reach around 118 ± 3 °C in 90 s (Figure S2), suggesting the heating performance was stable even using various batches of the composite paper.

The driven voltage of the heater may also influence the heating performance. As shown in Figure 3d, the maximum temperature of the heater gradually increased from 32.4 to 239.8 °C when the voltage increased from 1.5 to 9 V. Moreover, the maximum temperature and the power density as a function of the square of the voltage (U^2^) showed a good linear relationship (Figure 3e, f), suggesting that the resistance of the heater did not change in higher temperatures. That means the flexible heater possesses good thermal stability. Furthermore, the maximum temperature as a function of the power density is depicted in Figure 3g, which also appeared as a linear relationship. The slope of the fitting line was the power efficiency of 375.3 °C cm^2^ w^−1^, which is much higher than that of traditional indium tin oxide (ITO) glass (88 °C cm^2^ w^−1^) [34]. The working stability of the heater was finally measured under alternative voltage of 6 V. As shown in Figure 3g, the temperature of the heater can reach around 122.2 °C under power supply and then quickly falls down to around 25 °C. The temperature runs for 100 cycles without any observed attenuation, proving its good stability in repeat heating cycles. It should be pointed out that when the heater was heated to 239 °C, it was still stable since the thermogravimetry analysis suggested that it was stable blow 294 °C (Figure S3).

**Figure 4:**
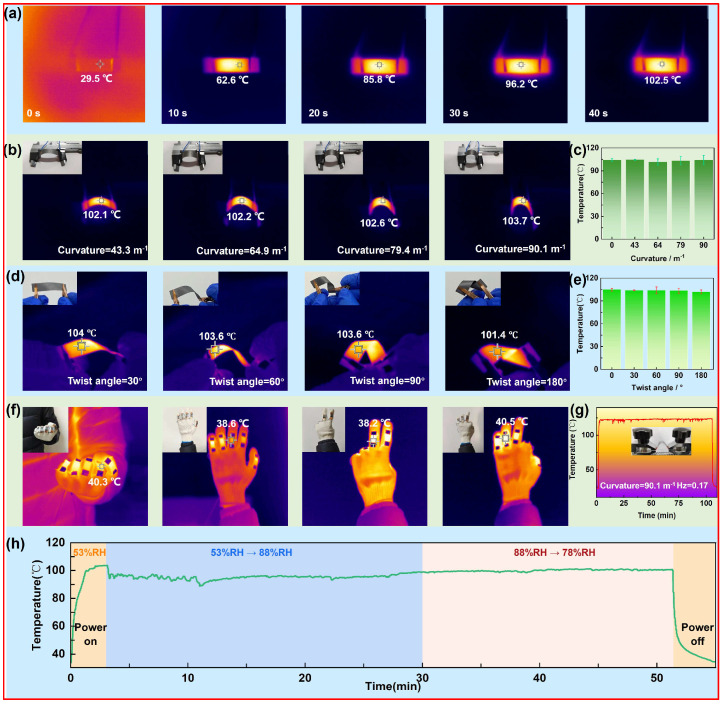
(**a**) IR images of the flexible heater under 6 V with the extension of time. (**b**) IR images and photograph and (**c**) temperature of the heater under different curvature of bending. (**d**) IR images and photograph and (**e**) temperature of the heater under twist angle. (**f**) IR images and photograph of the hand under the voltage of 3 V with gesture of clenching, opening, and gesturing “v” and “ok” when the glove was attached with the flexible heater on figures, and (**g**) temperature of the heater under 1000 bending cycles under strain of 0.5% with frequency of around 0.17 Hz, The red line indicates real-time temperature change. (**h**) Real-time temperature of the heater recorded when the relative humidity (RH) changed from 53 to 88%, The green line indicates real-time temperature change.

The electrothermal performance was then visualized by the infrared thermal imaging device. From the IR image of the heater under the voltage of 6 V (Figure 4a), it is found that the heater quickly turns red and reaches the maximum temperature of 102.5 °C within 40 s. When the bending strain (ε) was applied on the heater, the IR images in Figure 4b appeared unchanged, with the stable temperature at around 102 ± 2 °C (Figure 4b) when the curvature of the bending varied from 0 to 90.1 m^−1^, indicating that the bending strain has no significant impact on the electrothermal property of the heater. Furthermore, under twisting strain from 30 to 180° (Figure 4d), the temperature of the heater can also remain stable at around 103 ± 2 °C (Figure 4e), further proving its strain tolerance. A set of flexible heaters were then attached on the fingers of a glove to establish a wearable device (Figure 4f). Under the voltage of 3 V, all fingers were warmed up. The IR images were then recorded when the hand showed gesture of clenching, opening, and gesturing “v” and “ok”; the heaters appear almost uniform heating at the temperature of around 39 ± 1 °C (Figure 4f), indicating that the heater has good flexibility and acceptable stability under deformations. This result implied the heater may find application in wearable equipment with the function of thermal management. Moreover, the temperature of the heater was monitored in real time when the bending strain was alternatively applied on the heater with a curvature of 90.1 m^−1^ and a frequency of 0.17 Hz. From the temperature curve in Figure 4g, it is found that the temperature always remained around 120 °C in 100 min, further proving the good working stability of the flexible heater under deformation. Then, the impact of the relative humidity (RH) around the environment for the electrothermal performance was evaluated, as shown in Figure 4h. It is found that the temperature fluctuation of the heater may remain around 10% when the RH increases from 53 to 88% then decreases to around 78%, which may be caused by the hydrophilic feature of the paper-based materials. Therefore, the encapsulation of this heater may be necessary in real applications in complex conditions.

**Figure 5:**
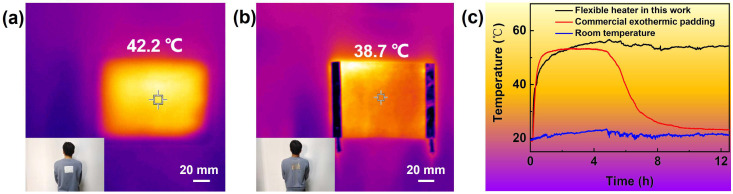
IR images and photographs of (**a**) commercial exothermic padding and (**b**) the flexible heater attached onto the back of human body and (**c**) the real-time monitoring of the temperature change within 12 h under 6 V with size of around 95 × 130 mm.

In order to explore the application of the heater as a wearable electric, the heating behavior of the commercial exothermic padding and the flexible heater in this work were evaluated and compared, for which the heater was cut into the same size as the commercial exothermic padding. It is found that the commercial exothermic padding appeared at a temperature of 42.2 °C (Figure 5a), while the flexible heater exhibited a temperature of 38.7 °C under the voltage of 6 V (Figure 5b), both of which gave warm feelings to the tester. Furthermore, the temperatures of them were monitored in real time for 12 h (Figure 5c). It is found that both of them can quickly reach the highest temperature soon; however, the temperature of the commercial exothermic padding significantly decreased after 5 h because the chemicals had run out. On the contrary, the flexible heater remained warm after 12 h. It should be noticed that the commercial exothermic padding usually can be used only one time and it may bring environmental impact after discarding. These results suggested that the flexible heater here may find application in wearable equipment.

**Figure 6:**
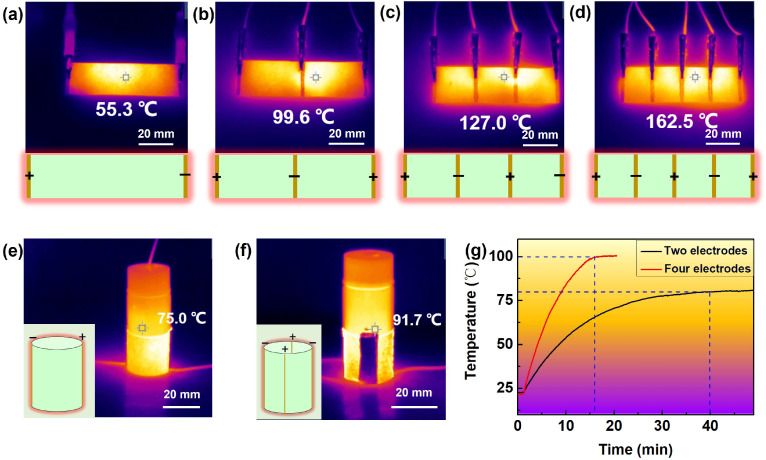
(**a**–**d**) IR images of the flexible heater with different numbers of electrodes. (**e**,**f**) IR images of the heater with two electrodes or four electrodes used for water heating and (**g**) the corresponding temperature change curve under 6 V with a size of 70 × 20 mm, The dashed lines indicate the time and temperature at which the maximum is reached.

As a heating device, the device structure may also influence the performance. Here, we found that the heating performance may be improved by increasing the number of electrodes. As shown in Figure 6a–d, the size of the flexible heater was 70 × 20 mm, various numbers of the electrodes were connected, and the maximum temperature was recorded and visualized with an infrared thermal imaging device. It is found that the highest temperature appeared as 55.3 °C, 99.6 °C, 127.0 °C, and 162.5 °C when the number of electrodes was 2, 3, 4 and 5, respectively. Furthermore, a bottle of 15 mL of water was heated with the device (Figure 6e,f) lasting for the same time; one may reach 75 °C with two electrodes and the other may target 91.7 °C with four electrodes. If the temperature of the water was recorded in real time, it was found that the water reached 100 °C in 16 min using the four-electrode heater but the water could only achieve 80 °C within 40 min using the two-electrode heater. It means that the heating behavior of the flexible heater may be regulated with the arrangement of the electrodes.

**Figure 7:**
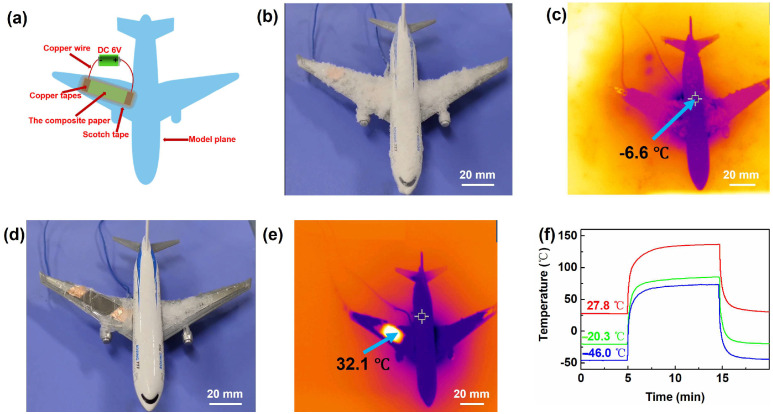
(**a**) Structure diagram of the model aircraft deicing using the flexible heater. (**b**) Photograph and (**c**) IR image of the model aircraft covered in snow. (**d**) Photograph and (**e**) IR images of the model aircraft during deicing with the heater. (**f**) Heating curve of the heater from different ambient temperatures under 6 V.

Aircraft icing is one of the most important inducements for aircraft accident [35]. The deicing treatment is the main solution to eliminate such danger. As a proof of the concept, the flexible heater was fixed on one wing of the model aircraft, as shown in Figure 7a. The aircraft was frozen in liquid nitrogen for five minutes then covered with snow (Figure 7b), showing the temperature was −6.6 °C in the IR image (Figure 7c). The heater was then driven under the voltage of 6 V, and the snow melted soon (Figure 7d). After 15 min, the temperature of the heater rose up to around 32.1 °C from the IR image (Figure 7e). To further confirm the working performance of the heater at low temperature, the heating curve of the device starting from different ambient temperatures was recoded as shown in Figure 7f. It is found that the heater can reach 137.2 °C after heating for 10 min when the starting ambient temperature is 27.8 °C, for which the temperature increment (∆*T*) was around 109.4 °C. By contrast, the heater can achieve 85.3 °C from −20.3 °C and target 73.5 °C from −46.0 °C, for which the ∆*Ts* were 105.6 °C and 119.5 °C, respectively. Obviously, the heater can work under low temperature. The maximum temperature may be different under various ambient temperatures, but the ∆*T* was comparable. This finding may provide guidance for the application of the flexible heater under low temperature.

## 4. Conclusions

In conclusion, a highly stable and bending-tolerant flexible heater was developed using renewable raw material of cellulose fibers composited with graphite nanosheets through a scalable papermaking technique. The electrothermal performance was improved by the hot-pressing treatment and the addition of CPAM. The heater can target a maximum temperature up to 239.8 °C in 1 min under the voltage of 9 V with a high power density of 375.3 °C cm^2^ w^−1^. It has shown good working stability over 100 alternatively heating and cooling cycles and even long-time heating lasting for 12 h. It appeared to have good bending tolerance under continuous bending deformations with various curvatures. It was proved to be used as a wearable heating equipment, soft heating device, and deicing for modal aircrafts, suggesting the versatile prospects in the application of heat management. In the future work, the water tolerance of this flexible heater may be improved by compositing with hydrophobic polymer materials or encapsulation.

## Data Availability

The original contributions presented in the study are included in the article/Supplementary Material, further inquiries can be directed to the corresponding author.

## Supplementary Materials

The following supporting information can be downloaded at: https://www.mdpi.com/article/10.3390/ma17143507/s1, Figure S1: The stress–strain curve of the heater before and after hot pressing; Figure S2: Temperature change curve of the flexible heater using (a,b) different sites of the one paper sample (116 ± 4 °C) and (c,d) different batches of the paper samples (118 ± 3 °C); Figure S3: TG and TGA curves of the flexible heater materials.
