# Supplementary material for: Highly Stable, Bending-Tolerant, and Sustainable Flexible Heater through a Scalable Papermaking Procedure
Source: Materials (Basel). 2024 Jul 15;17(14):3507. doi: 10.3390/ma17143507 (PMC11278561; doi:10.3390/ma17143507)
Supplement: Supplementary file 1 [file materials-17-03507-s001.zip › materials-3073381-supplementary.pdf]

Supporting Information

Highly Stable, Bending-Tolerant, and Sustainable Flexible Heater through a Scalable Papermaking Procedure

Jiajie Liu[a#], Huacui Xiang[b#], Wei Wang[b#], Xiujuan Tao[a], Zhou Bai[b], Zhijian Li[b], Haiwei Wu[b], Suochao Yuan[b], Hongwei Zhou[c], Hanbin Liu[b*]

[a] College of Chemistry and Chemical Engineering, Shaanxi University of Science & Technology, Xi'an, P.R. China (710021)
[b] Shaanxi Provincial Key Laboratory of Papermaking Technology and Specialty Paper Development, College of Bioresource Chemical and Materials Engineering, Shaanxi University of Science & Technology, Xi'an, P.R. China (710021)
[c] School of Materials and Chemical Engineering, Xi'an Technological University, Xi'an, P. R. China (710021)
[#]These authors contribute equally.
Corresponding Author
* liuhanbin@sust.edu.cn; hanbin_liu@foxmail.com (H. Liu).

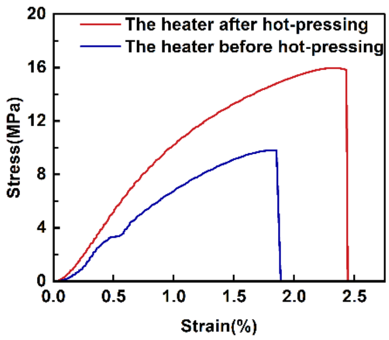

Figure S1. The stress-strain curve of the heater before and after hot pressing.

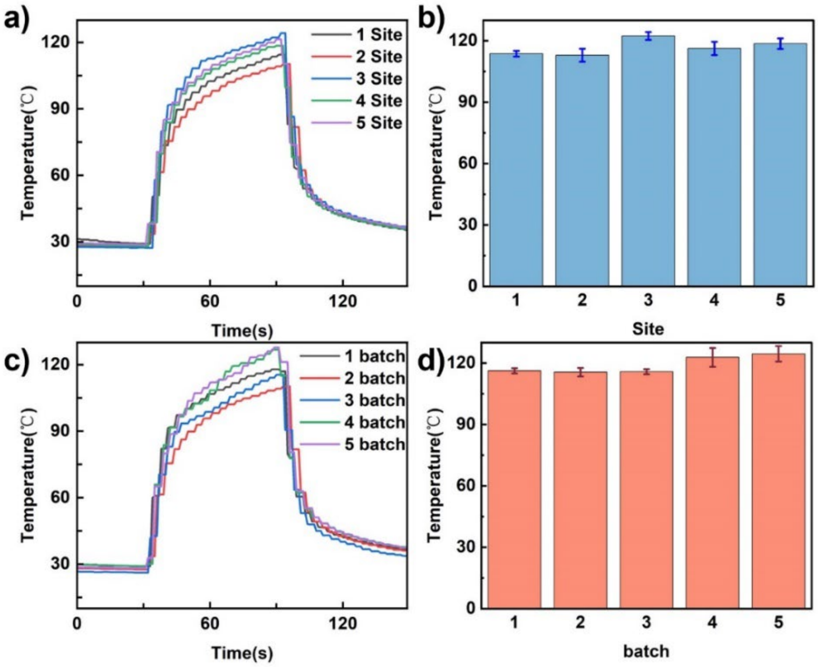

Figure S2. Temperature change curve of the flexible heater using (**a, b**) different site of the one paper sample (116±4 °C) and (**c, d**) different batch of the paper samples (118±3 °C).

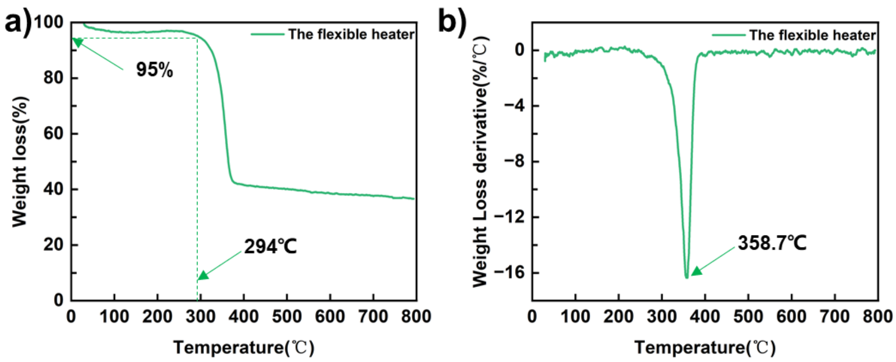

Figure S3. TG and TGA curve of the flexible heater materials.
